# Minimally Symptomatic Severe Hyponatremia: Two Case Reports

**DOI:** 10.5811/cpcem.47041

**Published:** 2026-03-10

**Authors:** Jordan Richardson, Neha Raukar, Luke Wood

**Affiliations:** * Mayo Clinic Alix School of Medicine, Department of Emergency Medicine, Rochester, Minnesota; † Regions Hospital, Department of Emergency Medicine, St. Paul, Minnesota

**Keywords:** hyponatremia, thiazide diuretic, diuretics, volume loss, case report

## Abstract

**Introduction:**

Hyponatremia is a common and often vexing electrolyte abnormality seen in the emergency setting. The severity of a patient’s symptoms is often dictated by the acuity of hyponatremia development and degree of serum sodium deficit, with patients typically demonstrating more severe neurological symptoms in acute-onset severe hyponatremia. Patients prescribed chlorthalidone are at particular risk of developing hyponatremia, especially in the setting of a secondary insult.

**Case Report:**

We describe two patients presenting to the emergency department with severe hyponatremia who were taking chlorthalidone. Both patients had clinical symptoms that were mild given the degree of their hyponatremia. Additionally, each patient had a secondary insult affecting their volume status that was an important contributing factor in the development of hyponatremia.

**Conclusion:**

Thiazide diuretic use, particularly chlorthalidone, is an important consideration when evaluating patients with new-onset hyponatremia, especially in the setting of excess volume losses or increased consumption of water. These cases demonstrate how patients with severe hyponatremia can present with atypical or mild clinical symptoms and often do not demonstrate significant neurological symptoms such as seizures.

## INTRODUCTION

Hyponatremia, defined as a serum sodium (SNa) concentration < 135 milliequivalents per liter (mEq/L), is one of the most common electrolyte abnormalities encountered in the emergency department (ED). It has a variety of underlying causes. The most frequent presentation is hypotonic hyponatremia, which can be sub-classified into three categories based on volume status: hypervolemic**;** hypovolemic**;** and euvolemic.^1^ Hypervolemic hypotonic hyponatremia occurs when water retention exceeds the body’s ability to excrete it, often due to kidney dysfunction, heart failure, cirrhosis, or nephrotic syndrome. Hypovolemic hypotonic hyponatremia results from sodium losses in the form of osmotic diuresis, adrenal insufficiency, vomiting, diarrhea, or excessive sweating. Euvolemic hypotonic hyponatremia is often associated with thiazide diuretic use, severe hypothyroidism, low solute intake, syndrome of inappropriate antidiuresis, or primary polydipsia.^1^

The symptoms of hyponatremia are varied and typically correlate to both the severity and acuity of the electrolyte derangement. Chronic hyponatremia can be asymptomatic or present with mild symptoms including disorientation or lethargy.^2^ Acute mild hyponatremia can present as nausea, vomiting, decreased appetite, or headache, which can progress to altered mental status, seizures, and coma. The risks of inadequate or delayed treatment, including cerebral edema and demyelination, are significant and life-threatening.^2^ In this report of two patients taking chlorthalidone who presented with mild symptoms despite severe hyponatremia, we discuss acute and chronic hyponatremia in the context of chlorthalidone use and share considerations for managing hyponatremia in the ED.

## CASE REPORTS

### Case 1

A 65-year-old female presented to the ED with the chief complaint of increased depression. Further history revealed a one-week history of intermittent vomiting, sleep difficulties, and reduced appetite. The patient’s husband presented with her and described episodic gait instability and vague speech difficulties over the prior two days. Her medical history included hypothyroidism, depression, and resistant hypertension, for which chlorthalidone 25 mg had been initiated 16 days prior. The patient denied any associated headaches, vision changes, syncopal episodes, or seizures.

Upon arrival, the patient was afebrile and hemodynamically stable. Cardiopulmonary exam was unremarkable, and she had no focal abdominal tenderness. Neurological exam demonstrated no cranial nerve deficits with full and symmetrical strength and gross sensation. She had no past-pointing, dysmetria, or abnormal heel-to-shin testing. No dysarthric speech or expressive aphasia was noted. Her gait was described as slowed but stable and non-ataxic. She denied any suicidal ideations but had a withdrawn affect and endorsed feelings of increased depression with poor sleep and appetite.

The patient’s husband reiterated the intermittent nature of her gait difficulties and speech changes, noting her normal bedside neurological exam. He once again expressed concern for her worsening depression. The emergency care team suspected either a potential neurological or psychiatric etiology. Her initial workup included complete blood count, basic metabolic panel, magnesium level, point-of-care venous blood gas, coagulation panel, thyroid function cascade, electrocardiogram, computed tomography (CT) head, and CT angiography of the head and neck with planned psychiatric consultation if the medical workup was unrevealing.

Prior to imaging, the patient had point-of-care venous blood gas demonstrating pH 7.51. Partial pressure of carbon dioxide was 34 millimeters of mercury (mm Hg) (reference range: 41–51 mm Hg), and SNa < 100 mEq/L (135–145 mEq/L). Given the patient’s mild, intermittent symptoms, non-focal neurological exam and SNa < 100, this laboratory finding was presumed to be in error and management of hyponatremia was deferred until the patient’s basic metabolic panel (BMP) had resulted. Basic metabolic panel returned showing SNa of 98 mEq/L and, once again, the accuracy of this lab result was questioned. It seemed unlikely for a hyponatremia of this severity to develop acutely (with normal SNa 16 days prior) in the absence of seizures or other objective neurological changes. A repeat BMP confirmed the presence of profound hyponatremia (SNa 97 mEq/L). The patient was also found to be hypomagnesemic to a level of 1.1 mg per deciliter (dL) (1.7–2.3 mg/dL). She was started on fluid restriction, and we obtained serum osmolarity, urine osmolarity, and urine sodium to classify her new-onset hyponatremia.

*CPC-EM Capsule*

What do we already know about this clinical entity?

*Hyponatremia can present as a myriad of clinical symptoms. Typically, the severity of symptoms relates to the degree of sodium deficit and whether the onset was acute or chronic*.

What makes this presentation of disease reportable?

*Acute, severe hyponatremia should result in significant clinical symptoms and objective neurological findings. Both cases demonstrate how this can present in patients with minimal symptoms*.

What is the major learning point?

*Thiazide diuretics predispose patients to developing acute hyponatremia. A secondary insult that affects volume status often predisposes these patients to severe hyponatremia*.

How might this improve emergency medicine practice?

*Hyponatremia can present with symptoms discordant from the degree of sodium deficit and timing of onset. Thiazide diuretic use is an important risk factor to be considered in these patients*.

The patient’s potassium and magnesium were repleted, and she received 50 mL of hypertonic saline in the ED. Chlorthalidone, a thiazide diuretic, was discontinued because of the known side effect of hyponatremia. She was transferred to the intensive care unit for further management, where she was found to have *Clostridium difficile* colitis contributing to her gastrointestinal (GI) symptomology and significant volume losses.

### Case 2

A 61-year-old male was referred to the ED after outpatient lab studies earlier in the day returned showing hyponatremia (SNa 116 mEq/L). Medications included metformin 2 grams, atorvastatin 20 mg, lisinopril 20 mg, and topical testosterone 40 mg, as well as chlorthalidone 25 mg for over five years. Given his metformin use, the patient had been encouraged to drink copious water in preparation for an outpatient abdominal CT for chronic nausea and diarrhea, and he had consumed four 20-ounce bottles of water. Following the patient’s CT, he drank an additional two 20-ounce bottles of water and then had blood work performed that revealed significant hyponatremia.

Upon arrival at the ED, the patient was hemodynamically stable and denied headache, confusion, syncope, seizures, dizziness, or weakness. The physical exam was unremarkable and non-focal. Lab work performed in the ED showed a SNa level of 116 mEq/L. Chlorthalidone was discontinued, and he was started on normal saline with free water restrictions. The patient was admitted to the hospital for further fluid and electrolyte management.

## DISCUSSION

These cases both illustrate profound hyponatremia in the setting of an acute change in volume status while prescribed chlorthalidone. In both cases, the patients had relatively mild symptoms disproportionate to the degree of sodium deficit. In Case 1, the patient had a SNa level of 97 mEq/L yet was reporting only poor sleep and appetite loss. Her husband had noted mild speech slurring and gait instability on a subacute basis. The patient in Case 2 was asymptomatic.

In this discussion, we review the literature on chlorthalidone and hyponatremia, presenting symptoms of chronic and acute hyponatremia, and important clinical considerations for hyponatremia in the ED. Hyponatremia is a well-known side effect of thiazide diuretics. Risk factors include advanced age, female sex, inappropriate osmotic diuresis, and mild hypokalemia.^3^ The time between thiazide diuretic initiation and hyponatremia can range from a few days to over a year with metabolic derangements typically presenting within 2–3 weeks of initiation of treatment.^4^ Thiazide diuretics act by blocking the resorption of sodium chloride in the distal convoluted tubule, thus inhibiting osmotic water resorption. However, thiazide diuretics have varying propensities to cause hyponatremia. Several studies have found that chlorthalidone is associated with a higher risk of hyponatremia than hydrochlorothiazide when used in equal doses.^5^–^7^

Hydrochlorothiazide has a less potent effect on blood pressure and is often prescribed at a higher dosage compared to chlorthalidone. When adjusting for this difference, one study found that the risk of developing hyponatremia was not significantly different in patients taking chlorthalidone (12.5–25 mg/day) compared to patients taking a two-fold higher dose per day of hydrochlorothiazide.^5^ Currently, guidelines are lacking regarding monitoring for electrolyte derangements when thiazide diuretics are initiated; therefore, patients can present with hyponatremia after recent initiation of treatment or with long-term use of chlorthalidone.

These two cases demonstrate that while we typically associate hyponatremia secondary to thiazide use with euvolemic hyponatremia, the presence of a secondary insult in volume status can make the hyponatremia even more severe. In our first case, the patient was hypovolemic secondary to *C difficile* infection with several days of preceding GI volume losses. Volume depletion can lead to increased release of antidiuretic hormone, resulting in the production of more concentrated urine.^4^ This can potentiate underlying hyponatremia from the diuretic with increased water retention, causing a dilutional effect with sodium and other electrolytes, thus resulting in a hypovolemic hypotonic hyponatremia.

In the second case, the patient had been instructed to increase his water intake significantly in the day leading up to his presentation, resulting in a dilutional hyponatremia. It has been noted that some patients with thiazide-induced hyponatremia have been found to have underlying polydipsia at baseline.^8^ In this case, the patient had hypervolemic hypotonic hyponatremia. Both patients were susceptible to hyponatremia due to their chlorthalidone use, but in both cases a secondary insult to their volume status was critical to the development of severe hyponatremia.

Hyponatremia can present as acute, subacute, and chronic. Acute hyponatremia, defined as hyponatremia that develops in < 48 hours, typically present with more pervasive and severe symptoms due to insufficient time for adaptive shifts in osmolality to protect from cerebral edema.^1^ Severe, acute hyponatremia can present with altered mental status, seizures, coma, and neurogenic shock.^9^ However, subacute or chronic hyponatremia, occurring over a period > 48 hours, is more often associated with milder symptoms of gait instability, dysarthria, disorientation, and somnolence, similar to the patient’s presenting symptoms in case 1.^2^

When evaluating hyponatremia in the ED, there are several important, specific considerations. To determine the etiology of hyponatremia, it is imperative to establish both volume status and osmolality to differentiate the type of hyponatremia, which ultimately guides clinical management. Point-of-care ultrasound, clinical exam, and serum creatinine can all be used to determine volume status.^9^ Osmolality, urine osmolality, and urine sodium should also be collected, with important consideration for collecting these labs before initiating any saline repletion. Additional considerations for the emergency physician include collecting serum glucose to rule out hyperglycemic hyponatremia, reviewing the patient’s medication list, and measuring serum potassium as this is also often concurrently depleted in hyponatremic patients. Emergency physicians should also be aware that there can be significant variations between point-of-care and standard laboratory measurements of SNa by up to 4 millimoles.^10^ While this may not be clinically meaningful for diagnostic purposes, it is important to ensure that the same method is being used for serial measurements during sodium repletion.

## CONCLUSION

We present two cases illustrating severe hyponatremia occurring in patients on chlorthalidone with secondary insults. Given the acuity and severity of their hyponatremia, both patients had relatively mild clinical symptoms, and neither presented with seizures or significant alterations to mental status. Chlorthalidone use is an important risk factor for developing hyponatremia, especially in patients who have a secondary insult affecting their volume status. These cases demonstrate how the emergency clinician must maintain a high degree of suspicion for potential hyponatremia when patients are prescribed chlorthalidone and are presenting with vague neurological symptoms. These cases also illustrate how patients can have profound hyponatremia and present asymptomatically or with mild clinical symptoms.
